# Personalized medicine: the future is here

**DOI:** 10.3325/cmj.2024.65.169

**Published:** 2024-06

**Authors:** Dragan Primorac, Aaron Ciechanover

**Affiliations:** 1 St. Catherine Hospital, Zagreb, Croatia; *dragan.primorac@svkatarina.hr*; 2 The Polak Cancer and Vascular Biology Research Center, Rappaport Faculty of Medicine and Research Institute, Technion-Israel Institute of Technology, Haifa, Israel

A rapidly evolving approach – personalized or precise medicine – aims to optimize diagnostics and treatment efficiency using patients’ molecular profiling. In modern medicine, we have never come closer to unraveling the underlying mechanisms of diseases at the molecular level. The new multi-OMICs approach firmly challenges the “one size fits all” canonical approach and promotes more accurate concepts. A significant shift from the old paradigm arose following the completion of the Human Genome Project and a considerable reduction in the cost of DNA sequencing. However, the complexity of the human genome, with about 19 370 genes and over 14 000 pseudogenes, is still a huge challenge. The parallel development of other Omics experimental tools – transcriptomics, proteomics, and metabolomics – and the involvement of other layers of regulation of gene expression, such as long non-coding and micro RNAs, and epigenetics add to this complexity. Nevertheless, expanding personalized medicine in clinical practice will put increased pressure on the health care system, particularly because of the lack of infrastructure and highly trained individuals. There also remain the challenges of handling and implementing a tremendous amount of research and clinical data into clinical practice.

AI systems have enabled us to analyze an enormous amount of real-time data in a cost-effective manner. Electronic health records as a platform play a key role in big data analysis, since they should eventually comprise patients’ multi-OMICS data and clinical findings. In this context, the term multi-OMICS is usually related to the comprehensive and multiple high-throughput systematic analysis of the epigenome, genome, metabolome, proteome, lipidome, glycome, transcriptome, etc. The multi-OMICS concept could enable us to identify new biomarkers or relevant signaling pathways important for disease development. Genetic biomarkers are becoming increasingly important in developing treatment or diagnostic algorithms, particularly in calculating genetic risk and assessing the treatment response. However, the multi-OMICS approach is not sufficient. It also needs analyses of the exposome (health effects of cumulative environmental exposures and concomitant biological responses), phenome (the sum of its phenotypic traits, skin color, height, eye color, etc.), and the patient’s clinical data using data integration algorithms. These additional data are essential for detailed disease analysis and treatment optimization. In the next decade, the “OMICS-based” approach will change the medicine we practice today, particularly diagnostics and treatment.

Recently, a dramatic shift has happened with the introduction of single-cell omics. Surprisingly, single-cell omics revealed a significant heterogeneity of genomics, transcriptomics, and epigenomics, dramatically challenging our current concept of treatment, particularly in cancer. On the other hand, the spatial transcriptome (ST) enables us to analyze the multi-OMICS of multiple tissues simultaneously. For example, the ST, as part of multi-OMICS, can facilitate the creation of a tumor cell map, which can be critical for generating targeted therapy and calculating overall survival. Knowing the importance of single-cell OMICS, it is critical to understand the complexity of the cellular structure. Roughly, we are built of 30 trillion cells (3 × 10^13^), while 300 billion cells (3.0 × 10^11^) are replaced daily. Accordingly, about every three months, we entirely restore ourselves. Besides that, ~ 5%-8% of our proteins are daily degraded and replaced.

DNA stability is critical for all the physiological processes in our body. However, each of our cells could suffer more than a million mutations per day, and any defect in the DNA repair mechanism could be potentially hazardous. Additionally, the human body consists of 38 trillion (3.8 × 10^13^) bacteria that constantly interact with our cells. Undoubtedly, utilizing the microbiome as a part of personalized medicine is knocking on our door. The individual human microbiome “fingerprint” is unique and influenced by endogenous factors (particularly host genetics and immunity), exogenous factors, lifestyle, environment, etc (1). Recent data suggest that changes in the human microbiome over time correlate with numerous markers responsible for cardiometabolic diseases (2).

For years, personalized medicine and DNA analysis in clinical work have been based on germline mutation detection. However, much less is known about somatic (acquired) mutations. Germline mutations could be present in all somatic cells, while somatic mutations are more or less specific for a post-zygotic cell population. The somatic mutation rate is almost two orders of magnitude higher than the germline mutation rate (3). In cancer with “two different genomes,” the situation is even more complex. Testing for somatic mutations (tumor testing) and microsatellite instability, usually through a biopsy or surgery, becomes critical in determining targeted therapy for certain tumors. The therapeutic benefits of targeted therapy for specific mutations are directly correlated with treatment success, particularly in breast cancer (targeting human epidermal growth factor [HER2]) and in lung cancer (targeting epidermal growth factor receptor [EGFR] related to tyrosine kinase inhibitor response) (4).

An emerging technology, liquid biopsy, detects cell-free or circulating tumor DNA (ctDNA) released by tumor cells in the blood and eventually in urine, saliva, cerebrospinal fluid, etc. ctDNA, another key component of understanding the mutation landscape of malignant diseases, serves as a useful tool in optimizing targeted therapies, including immunotherapy. Besides, ctDNA analysis could provide information about the optimal therapeutic targets, potential drug resistance, and tumor evolution over time. Additionally, the liquid biopsy approach is less invasive than tissue biopsy (which is not always possible, eg, in the brain) and it allows us to follow the treatment results in real time (5).

The recent introduction of whole genome sequencing (WGS) into clinical practice has become a game-changer, as detecting DNA polymorphisms in both exons and introns is critical to understanding disease predisposition and drug response. At the same time, together with DNA analysis, WGS helps study miRNA (having an important regulatory function), small nuclear RNA, ribosomal RNA, transfer RNA, etc. By using WGS for the first time, we systematically enter the non-coding region of the genome, thus shedding light on the mechanism of many uncommon medical conditions and rare diseases. Nevertheless, by analyzing mutations in the regulatory elements within non-coding regions that could influence changes in gene expression, we are becoming more and more aware of their significant impact on phenotypic manifestation and disease development. However, since many clinical algorithms are based on whole exome sequencing, it took some time to develop AI-based algorithms capable of optimizing machine learning and deep learning models for clinical implementation (6). Specific areas of WGS utility in medicine involve cancer genomics, infectious disease diagnostics, pharmacogenomics, rare disease diagnostics, and others. Furthermore, WGS provides enormous amounts of data for every sequenced patient. With the use of AI tools, the potential usefulness of these data are endless. However, when approaching the topic of genomic sequencing in modern medicine, third- and fourth-generation technologies cannot be overlooked. These include long-read and hybrid-read sequencing for higher-precision and novel technologies such as nanopore sequencing (which appears to be applicable also to proteomic analyses). As sequencing is continuously becoming more accessible and cost-effective, its further integration into clinical practice seems inevitable.

A new concept for using WGS is newborn screening, which can become an essential tool for disease prevention and treatment optimization at an early age (7). Driven by cost reduction of WGS, new AI-based algorithms, and awareness that WGS could significantly improve treatment outcomes, population-based genetic screening interventions will likely become a reality within the next few years.

According to many, pharmacogenomics (PGx) is a key component of the personalized medicine concept of single nucleotide polymorphisms (SNPs) (8). SNPs make the difference in observed drug effects by modulating the activity of their protein product (metabolizing enzymes, transporters, drug-receptors, or other proteins not directly related to the drug) (9).

GnomAD, an online resource of genome sequencing data, contains allelic and genotype frequencies of drug-metabolizing enzymes for specific populations, helping the scientific community and physicians optimize the treatment. Even if the patient’s reaction to the drug is related to age, renal and liver functions, drug-drug interaction, and drug-food interaction, genetic factors alone account for up to 95% of individual drug responses (10). In 2009, the International Clinical Pharmacogenetics Implementation Consortium (CPIC) was established to implement PGx research data in the clinic. CPIC issued four categories of recommendation: A, B, C, and D. Category A, based on strong or moderate evidence, recommends dose adjustment or alternative drug; category B does the same but with conflicting and weaker evidence; while categories C and D of drug/gene association do not have sufficient evidence for pharmacogenomic testing.

Gene editing technologies, including clustered regularly interspaced short palindromic repeats and CRISPR-associated protein 9 (CRISPR-Cas9), will likely redefine the margins of the original personalized medicine concept, while monogenic diseases will most likely be the first targets in correcting pathogenic mutations. Still, a tremendous amount of energy must be invested in order to solve the complex issue of cell-specific delivery. On the other hand, since gene-editing technologies could alter cell/DNA in humans, it is necessary to develop a clear framework and policies related to gene and cell therapy ethics. Emerging RNA editing is already believed to be an alternative to gene editing.

Cell therapy is already taking a significant place in the personalized medicine concept, particularly in patients with cancer. For example, in the cancer immunotherapy approach, different modalities of autologous cell therapy, including the use of tumor-infiltrating lymphocytes, gene-modified T-cell receptor, or chimeric antigen receptor (CAR), for tumor treatment are becoming standard procedures. Yet, we are still learning about the complications of these procedures and the risks they carry. Thus, the United States Food and Drug Administration has recently warned against the cancer-inducing risk of the CAR-T treatment due to changes in the DNA of the treated T cells. However, it is becoming clear that the microenvironment significantly contributes to the treatment outcome (11). We witnessed a similar principle while treating patients with osteoarthritis with micro-fragmented fat tissue consisting of mesenchymal stem cells (12). Introducing WGS into oncology could significantly contribute not only to the assessment of the patient’s risk for developing cancer but also to targeting relevant biomarkers or genes for a certain cancer type. One example includes treatment with imatinib targeting the BCR-ABL1 fusion oncogene, consequently inhibiting tyrosine kinases in chronic myeloid leukemia patients, or treatment with poly-ADP ribose polymerase inhibitors targeting the BRCA1 and BRCA2 mutations in breast cancer patients (6).

Other therapeutic approaches based on the personalized medicine concept in oncology include immune checkpoint inhibitors, which target programmed death protein 1 (PD-1) and its ligand 1 (PD-L1), and cytotoxic T-lymphocyte associated protein 4. While PD-1 is expressed by immune cells, PD-L1 is expressed by cancer cells. Treatment with an immune checkpoint inhibitor such as pembrolizumab blocks PD-1 to prevent cancer from usurping the PD-1/PD-L1 pathway to escape the immune system (13).

There is tremendous potential in the new concept of “personalized regenerative medicine” focused on mesenchymal stem cells, known for their ability to treat tissue defects and regulate immune responses (14,15). Similarly, the emerging field of tissue engineering shows remarkable success in producing artificial tissue or organs, particularly the skin, cartilage, bladder, bone, etc. Attempts to produce more complex organs consisting of intricate three-dimensional vascular structures failed. To do so, we will need to better understand the cell biological processes involved in organogenesis, including their environment and the signals needed for functional behavior.

There are important ethical issues related to personalized medicine. One is pricing and accessibility. It is expected that each “disease” will be sliced and stratified into several driving mechanisms, and each will require the development of its own treatment. For example, we currently know four different causes for breast cancer – mutations in the *EGFR*, *HER2*, estrogen and progesterone receptors, and cancer that originates from none of the above, known as triple negative. New ones will probably be discovered as well. The same is probably true for Parkinson’s disease with mutations in several genes – *PRKN*, *SNCA*, *PINK1*, and *LRRK*, among others. While these and many other diseases have been treated with blockbuster drugs, the unraveling of their underlying mechanisms and the ability to identify them will result in the development of targeted therapies and shrinkage of the market of the blockbuster drugs. Needless to say, pricing will increase, as the share market of each drug will be smaller than that of the “one size fits all” drugs.

Another ethical aspect is the possible leakage of patients’ molecular information. As DNA sequencing, but also, in the future, additional important OMIC information, will be available for each patient, the leakage of this sensitive information or its hacking from medical facilities' computers may endanger patients’ privacy and make them vulnerable to blackmail. An important issue under discussion is whether we – or other formal agencies such as governments, employers, insurance, and health care providers – will have the motivation or interest to access this information. For the patients themselves, this information can be important for preventing diseases by knowing ahead of time that they carry a gene with a certain risk for a certain disease development (for example, *BRCA1*, mutated p53, or *RAS*). Yet, the testing can identify mutated genes for diseases for which we have no treatment. One example is the APOEϵ4 variant of the *APOE4* gene family, which carries a high risk for the development of Alzheimer disease. Disclosing its presence to a patient many years before the disease displays its first symptom (the development of the disease is not certain anyway), has serious family, employment, health insurance, and certainly emotional implications.

As we see, personalized medicine is not only personal or precise but also has a predictive power and, consequently, preventive power for some diseases. Importantly, it converts the physician from being the almighty authority to a professional consultant, and the patients to participants in the decision-making process. These 4Ps (personalized or precise, predictive, preventive, and participatory, as coined by Leroy Hood) have the potential to shake the three “canonical” pillars of medicine – the patient, the disease, and the treatment. With the ability to pre-diagnose a disease much before it erupts symptomatically, the patient can be the sperm, egg, fertilized egg, or embryo in its early stage of development. The disease can be a mutation – far from the clinical disease that develops decades down the road and therefore far from the sick patient in the hospital, and the treatment in these cases can be gene editing rather than a drug, surgery, irradiation, or cell therapy.

All these ethical issues require extensive societal discussions, and their results may depend on religious, legislative, historical, and political considerations prevailing in different societies and countries.
